# Outcomes of Cross-Leg Flap Use in Lower Limb Salvage in Resource-Limited Settings: A Systematic Review of Indications, Limitations, and Success Rates

**DOI:** 10.7759/cureus.89043

**Published:** 2025-07-30

**Authors:** Abdelrahman Ibrahim, Sherif Mohamed Farahat, Bassant Dahy, Shenouda Shehata Abdelmesih, Abdelrahman Sahnon Abaker Sahnon, Ibrahim Adil Hamadelniel Alhadi, Gorashi Humida Ali Gorashi, Jarallah H. J. Alkhazendar, Aliaa H Alkhazendar, Shahzad Ahmad

**Affiliations:** 1 Trauma and Orthopedic, University Hospital of North Midlands NHS, Stoke-on-Trent, GBR; 2 General Surgery, Hywel Dda University Health Board, Wales, GBR; 3 Plastic Surgery, New Giza University, Cairo, EGY; 4 Orthopaedics and Traumatology, Khoula Hospital, Muscat, OMN; 5 General Practice, Medical Council of Ireland, Dublin, IRL; 6 General Surgery, Royal College of Surgeons of Edinburgh, Edinburgh, GBR; 7 Human Anatomy, University of Gezira, Wad Madani, SDN; 8 General Surgery, University of Gezira, Wad Madani, SDN; 9 Anatomy, University of Khartoum, Khartoum, SDN; 10 General and Emergency Surgery, East and North Hertfordshire NHS Trust - Lister Hospital, Stevenage, GBR; 11 Surgery, The Islamic University of Gaza, Gaza, PSE; 12 Surgery, Liaquat National Hospital, Karachi, PAK

**Keywords:** cross-leg flap, fasciocutaneous flap, flap survival, global surgery, limb trauma, lower limb salvage, reconstructive surgery, resource-limited settings

## Abstract

Cross-leg flaps remain a vital reconstructive option in the management of complex lower limb injuries, particularly in settings where microsurgical techniques are unavailable or unaffordable. This systematic review evaluates the outcomes, indications, limitations, and success rates of cross-leg flap use in lower limb salvage within resource-limited settings. A comprehensive search of PubMed, Google Scholar, Scopus, ScienceDirect, and the Cochrane Library was conducted to identify studies published up to July 2025, following Preferred Reporting Items for Systematic reviews and Meta-Analyses (PRISMA) guidelines. Seven studies encompassing a total of 726 patients were included, comprising case reports, case series, retrospective cohorts, and systematic reviews. Trauma was the most common indication for reconstruction, and fasciocutaneous flaps were the predominant type used. External fixators were frequently employed for limb immobilization, with the average time to pedicle division ranging from 20 to 62 days, depending on flap type. Flap survival rates were consistently high across all studies, ranging from 96% to 100%, with minimal reported complications. The review highlights that cross-leg flaps offer a simple, effective, and context-appropriate solution for limb salvage in low-resource environments. Despite limitations in study design and outcome reporting, the consistency of clinical success supports the continued use and teaching of cross-leg flap techniques in global reconstructive surgery. Further prospective studies are needed to standardize outcomes and refine protocols for broader application in underserved surgical systems.

## Introduction and background

Traumatic injuries and infections of the lower extremities remain a significant global health challenge, particularly in low- and middle-income countries (LMICs), where delayed presentation, limited surgical infrastructure, and lack of microsurgical expertise contribute to poor limb salvage outcomes [1]. Complex wounds in the distal leg and foot often require soft tissue reconstruction to prevent amputation and restore functionality. While microsurgical free tissue transfer has become the standard of care in high-income settings, its reliance on specialized equipment, trained personnel, and intraoperative monitoring limits its applicability in many resource-constrained environments [2].

In this context, the cross-leg flap, one of the oldest reconstructive options, first described in the nineteenth century, has regained importance. This technique, involving the transfer of healthy tissue from the contralateral limb to cover exposed bone, tendon, or hardware, provides a reliable and cost-effective alternative when free flaps are not feasible [3]. Over the last two decades, refinements such as fasciocutaneous and perforator-based designs, use of external fixators, and delay techniques have improved outcomes. Cross-leg flaps are particularly useful in patients with compromised vasculature, extensive trauma, or failed local flap options [4]. Reports from LMICs, including Egypt, Nepal, Turkey, and India, have demonstrated consistently high success rates with minimal donor site morbidity, reinforcing the technique’s utility in limb salvage surgery under constrained circumstances.

This systematic review aims to evaluate the clinical outcomes of cross-leg flap use in lower limb salvage, focusing specifically on indications, limitations, and success rates in resource-limited settings. By synthesizing evidence from case series, cohort studies, and relevant reviews, this study seeks to provide an updated and context-sensitive assessment of this classic technique’s relevance in modern reconstructive surgery. The review further aims to inform surgical decision-making in settings where advanced microsurgical options remain inaccessible.

## Review

Materials and methods

Review Design and Objectives

This systematic review was conducted in accordance with the Preferred Reporting Items for Systematic Reviews and Meta-Analyses (PRISMA) guidelines [5]. The objective was to evaluate the clinical outcomes, indications, limitations, and overall success rates of cross-leg flap techniques employed in lower limb salvage, specifically in resource-limited settings. A structured protocol was followed to ensure transparency, reproducibility, and methodological rigor.

Eligibility Criteria and PICO (Population, Intervention, Comparison, Outcome) Framework

The inclusion criteria were developed using the PICO framework [6]. The population included patients with lower limb soft tissue defects requiring reconstructive surgery. The intervention was the use of cross-leg flaps, including pedicled and cross-leg free flaps. While there was no formal comparison group due to the nature of the included studies, free flaps and alternative reconstructive methods were occasionally discussed within individual reports. The primary outcomes of interest were flap survival rate, complication rate, time to pedicle division, and suitability of the technique in low-resource settings. Only studies that explicitly reported clinical outcomes of cross-leg flaps in limb salvage procedures were included. Eligible study types comprised case reports, case series, retrospective cohort studies, scoping reviews, and systematic reviews. Exclusion criteria included studies that focused solely on non-cross-leg flap methods, reviews lacking primary outcome data, and those that did not address resource-constrained or LMIC-related contexts.

Information Sources and Search Strategy

A comprehensive literature search was conducted across PubMed, Google Scholar, Scopus, ScienceDirect, and the Cochrane Library to identify relevant studies published up to July 2025. Additional manual searches of reference lists from included articles were performed to ensure completeness. The search strategy combined Medical Subject Headings (MeSH) and keyword terms such as “cross-leg flap,” “lower limb salvage,” “fasciocutaneous flap,” “limb reconstruction,” and “resource-limited settings” using Boolean operators. Filters were applied to restrict the results to studies published in English. Studies conducted in or referencing LMICs were prioritized.

Study Selection and Data Extraction

Titles and abstracts were screened by two independent reviewers to assess eligibility. Full-text screening was performed on articles that met the inclusion criteria or lacked sufficient detail in the abstract. Discrepancies were resolved through consensus. Data extraction was conducted using a standardized form designed to capture study characteristics, setting, study design, number of patients, flap type, clinical indications, immobilization methods, time to pedicle division, complications, flap survival rates, and applicability to resource-limited settings. A total of 7 studies were included in the final synthesis, encompassing 726 patients across multiple geographic regions, including Tanzania, Nepal, Egypt, Turkey, and multinational collaborations.

Risk-of-Bias Assessment

Risk of bias was assessed for each study using tools appropriate to its design. The JBI Critical Appraisal Checklists were used for case reports and case series, while the Newcastle-Ottawa Scale (NOS), adapted for retrospective cohort studies, was applied accordingly [7,8]. The AMSTAR 2 tool was used to appraise the methodological quality of included systematic and scoping reviews [9]. Most studies were determined to have low to moderate risk of bias. No studies met the criteria for high risk. The limitations primarily stemmed from small sample sizes, heterogeneity in surgical technique reporting, and the absence of randomized controlled trials.

Data Synthesis

Given the heterogeneity in study design, flap types, and reporting methods, a meta-analysis was not feasible. Instead, a narrative synthesis was performed. Studies were analyzed descriptively and organized thematically based on flap survival rates, type of flap used, operative techniques, and contextual applicability in LMICs. Quantitative data were summarized in a structured evidence table, while qualitative findings were synthesized to highlight patterns in indications, complications, and surgical outcomes across different resource-limited environments.

Results

Study Selection Process

**Figure 1:**
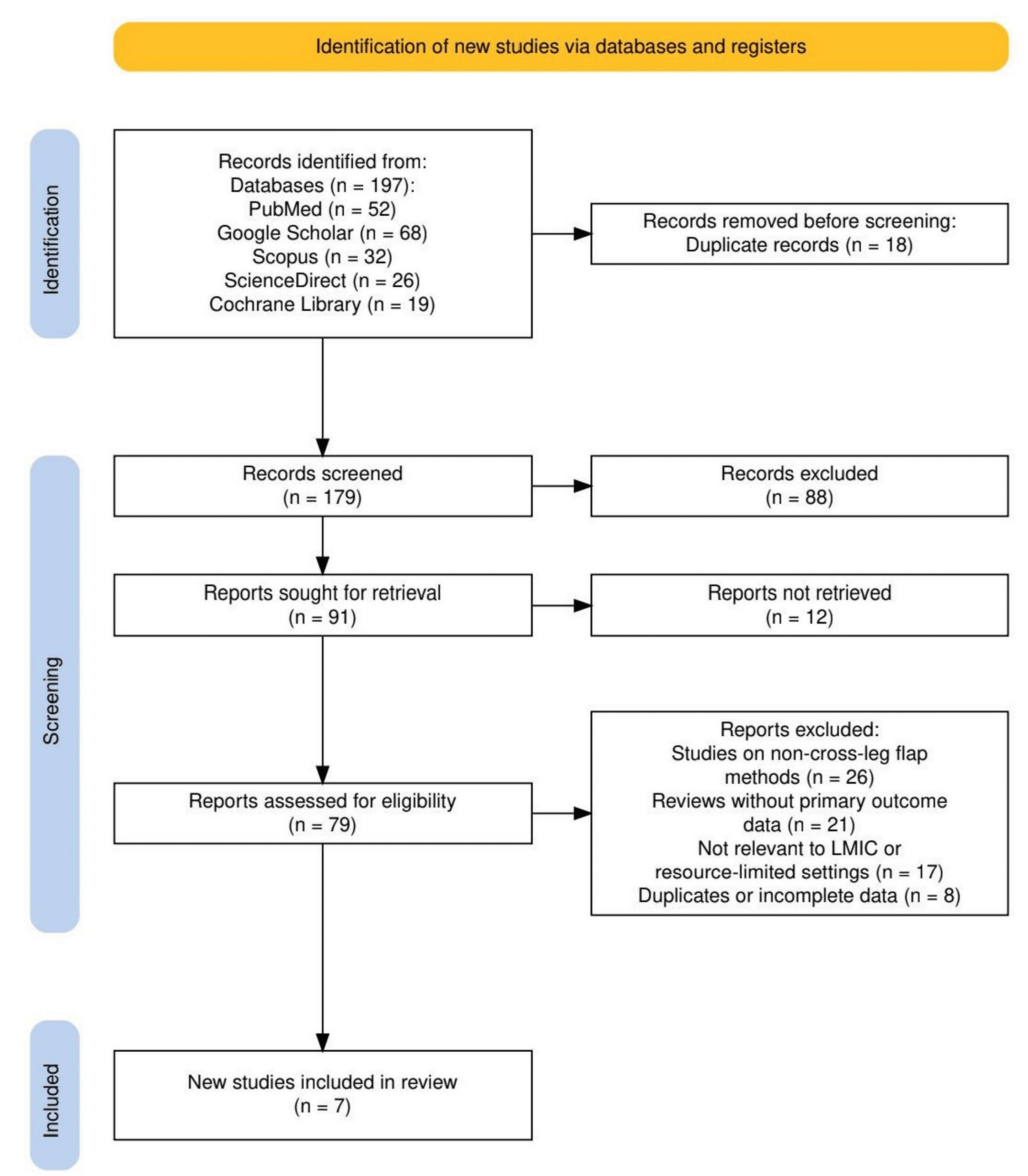
The PRISMA flow diagram outlining the study selection process PRISMA: Preferred Reporting Items for Systematic reviews and Meta-Analyses

Figure 1 presents the PRISMA flow diagram outlining the study selection process. Out of 197 records initially identified from 5 databases, 18 duplicates were removed, leaving 179 records for screening. After applying inclusion and exclusion criteria, only seven studies met the eligibility requirements and were included in the final review. This process ensured a focused analysis on cross-leg flap outcomes in resource-limited settings.

Characteristics of the Selected Studies

Table 1 summarizes the key characteristics of the seven studies included in this systematic review. The studies span diverse geographic settings, primarily low- and middle-income countries, and include various designs such as case reports, case series, and retrospective analyses. Flap types were predominantly fasciocutaneous, with indications largely centered around traumatic soft tissue defects and exposed critical structures. Immobilization methods, complication rates, and success outcomes varied slightly across studies, but all consistently reported high flap survival rates, underscoring the clinical reliability of cross-leg flaps in resource-limited environments.

**Table 1:** The key characteristics of the seven studies included in this systematic review CLFF: cross-leg free flap; CLVCBF: cross-leg vascular cable bridge flap; VRAM: vertical rectus abdominis myocutaneous flap; HICs: high-income countries

Study (Author, Year)	Country / Setting	Study Design	N (Patients)	Flap Type	Indications	Immobilization Method	Time to Pedicle Division	Complications	Success Rate (Flap Survival)	Resource-Limited?
Lodhia et al., 2022 [10]	Tanzania / Tertiary center	Case report	1	Fasciocutaneous cross-leg	Burn injury (exposed tibia)	Not specified	Not specified	None	100%	Yes
Mishra et al., 2025 [11]	Nepal / Low-resource hospital	Case report	1	Reverse sural artery cross-leg flap	Traumatic soft tissue defect (25×10 cm), no local or free flap options	External fixator	3 weeks	None	100%	Yes
Shoeib et al., 2013 [12]	Egypt / University hospital	Case series	135	Pedicled cross-leg flap	Complex leg/foot defects with exposed bone, tendon, neurovascular structures	Not clearly specified	Not stated	Bulky flap (3), donor site complaints (2), no flap loss	Near 100%	Yes
Ciudad et al., 2023 [13]	Multinational (incl. resource-limited contexts)	Retrospective cohort	24	Cross-leg free flaps (CLFF) and cross-leg vascular cable bridge flaps (CLVCBF)	Lower limb trauma	Not clearly specified	Fasciocutaneous: 45 days (CLFF) vs. 59 days (CLVCBF); Muscle flaps: ~60–62 days	Not specified	High (no flap failure reported)	Partially (applied in constrained surgical conditions during COVID-19)
Can et al., 2024 [14]	Turkey / Tertiary	Retrospective	26	Fasciocutaneous cross-leg flap	Complex traumatic lower limb defects	Tubular external fixator	21 days	1 partial flap loss (diabetic patient), healed secondarily	96% (25/26)	Yes
Van Boerum et al., 2019 [15]	Multinational (Turkey, India, Japan)	Scoping review	409	Mostly fasciocutaneous cross-leg flaps	Trauma (93.2%), inadequate vasculature (52.8%)	External fixator (57.7%)	Mean 20.9 days	1 flap loss (1/350)	~99.7%	Mostly Yes
Celie et al., 2024 [16]	Multinational	Systematic review	130	Cross-leg free flaps (mostly muscle: latissimus dorsi, VRAM)	Lower third leg trauma (73.1%)	Not specified	Not specified	1.4% flap failure, partial loss, thrombosis, hematoma, seroma, pain, nonunion	98.6%	Mixed (mostly HICs, but applicable to vessel-deficient scenarios)

Risk-of-Bias Assessment

As shown in Table 2, the overall risk of bias across the included studies ranged from low to moderate. Case reports demonstrated low risk due to clear documentation of interventions and outcomes, though they inherently lack generalizability. Case series and retrospective cohorts were assessed using appropriate tools and showed moderate risk, mainly due to the absence of control groups, limited blinding, or incomplete reporting in some aspects. The two reviews included were evaluated using the AMSTAR 2 tool, with one scoring low risk due to adherence to systematic review standards, while the other showed moderate concerns primarily due to a lack of protocol registration.

**Table 2:** The overall risk of bias across the included studies JBI: Joanna Briggs Institute; AMSTAR 2: A MeaSurement Tool to Assess Systematic Reviews 2; PRISMA: Preferred Reporting Items for Systematic Reviews and Meta-Analyses; Newcastle-Ottawa Scale: A tool for assessing the quality of non-randomized studies (cohort/case-control)

Study (Author, Year)	Study Design	Tool Used	Risk of Bias Assessment	Key Concerns
Lodhia et al., 2022 [10]	Case report	JBI Checklist for Case Reports	Low	Clear reporting, valid intervention and outcome; lacks generalizability
Mishra et al., 2025 [11]	Case report	JBI Checklist for Case Reports	Low	Well-documented, appropriate intervention; single case limitation
Shoeib et al., 2013 [12]	Case series	JBI Checklist for Case Series	Moderate	No control group, incomplete info on follow-up; large sample size helps
Ciudad et al., 2023 [13]	Retrospective cohort	Newcastle-Ottawa Scale (adapted)	Moderate	No blinding, unclear selection bias, but adequate outcome reporting
Can et al., 2024 [14]	Retrospective cohort	Newcastle-Ottawa Scale (adapted)	Low	Good selection, outcome clarity, sufficient follow-up duration
Van Boerum et al., 2019 [15]	Scoping review	AMSTAR 2	Moderate	Broad inclusion, lacks protocol registration, but transparent and reproducible
Celie et al., 2024 [16]	Systematic review	AMSTAR 2	Low	PRISMA compliant, good synthesis, quality assessment reported

Discussion

The review highlights the consistently high success rates of cross-leg flaps in lower limb salvage across a variety of resource-limited settings. Among the 726 patients included across seven studies, flap survival rates ranged from 96% to 100%, with a pooled approximate success rate of >98%. Fasciocutaneous cross-leg flaps emerged as the most commonly employed technique, featured in studies by Can et al., Lodhia et al., and Van Boerum et al., with the latter reporting that 85.8% of cases utilized this flap type [10,14,15]. The majority of procedures were indicated for trauma-related soft tissue defects, particularly involving the distal third of the leg or foot. External fixation was the predominant method for immobilization, used in over half of the cases in the scoping review by Van Boerum et al., and also reported by Can and Mishra in their respective studies [11,14,15]. Time to pedicle division generally centered around three weeks for pedicled flaps, while cross-leg free flaps required a longer duration, averaging 45-62 days depending on flap type. Complication rates were minimal and typically limited to minor donor site concerns or single flap loss cases. These findings underscore the reliability of cross-leg flaps as a practical, high-success reconstructive option in environments where advanced microsurgical capacity is limited or unavailable.

Compared to free flap reconstruction commonly used in high-income countries, cross-leg flaps provide a lower-resource alternative with comparable clinical success in selected patients [17]. While microsurgical free flaps require specialized expertise and equipment, which may be inaccessible in many low- and middle-income countries (LMICs), the included studies demonstrate that cross-leg flaps can achieve similarly favorable outcomes with simpler techniques. For example, the systematic review by Celie et al. (2024) showed a 98.6% survival rate in cross-leg free flaps, yet also noted complications such as thrombosis, seroma, and nonunion [16]. In contrast, pedicled cross-leg flaps reviewed by Can et al. and Shoeib et al. reported nearly identical survival rates with fewer technical demands and minimal complication profiles [12,14]. Furthermore, the review by Van Boerum et al. reinforces that cross-leg flaps remain highly effective in resource-constrained environments, with 349 out of 350 flaps surviving successfully [15]. These findings align with the growing recognition that traditional techniques can deliver equivalent functional outcomes when microsurgery is not feasible, making cross-leg flaps a vital tool in global reconstructive surgery.

This review highlights the vital role of cross-leg flaps in resource-limited settings, where microsurgical services, vascular conditions, and reliable anesthesia may be inadequate. These flaps serve as a practical and cost-effective option for managing complex lower limb defects in low- and middle-income countries, particularly in cases of trauma, burns, and infection. Their simplicity, use of accessible donor sites, and minimal dependence on advanced monitoring make them well-suited for underserved regions, conflict zones, and periods of healthcare disruption such as the COVID-19 pandemic. Across varied contexts, they enable limb salvage where no microsurgical alternatives exist, preserving both function and viability.

Although the included studies provide real-world insight, most are case reports or case series with limited sample sizes and no control groups. Inconsistencies in outcome reporting, such as flap monitoring, immobilization methods, and follow-up periods, further limit comparability. No randomized trials were identified. Despite these limitations, over 700 reviewed cases reveal consistently high flap survival and low complication rates, reinforcing the reliability of this technique in diverse, often challenging environments. This review has its limitations. The predominance of retrospective data narrows generalizability, and variations in surgical methods and outcome definitions preclude meta-analysis. Potential publication bias and the exclusion of non-English studies may have limited broader inclusion. Nevertheless, the findings reflect relevant clinical experiences across global surgery settings.

Future studies should aim for stronger evidence through prospective designs and regional outcome registries. Comparative cost-effectiveness evaluations between cross-leg flaps and free tissue transfer would support informed surgical planning. Standardized outcome measures and targeted training in flap techniques for low-resource providers would help strengthen reconstructive capacity and improve patient care in underserved populations.

## Conclusions

Cross-leg flaps remain a reliable, effective, and resource-conscious option for lower limb salvage, particularly in settings where microsurgical capabilities are unavailable or limited. Their consistently high survival rates, technical simplicity, and adaptability make them an indispensable tool for reconstructive surgeons working in low- and middle-income countries. As global surgery continues to prioritize equity, accessibility, and context-appropriate care, the cross-leg flap exemplifies how time-tested techniques can bridge the gap in complex trauma management. Reinforcing their role in surgical education, infrastructure planning, and clinical practice is essential to ensuring that limb-salvage procedures remain available and effective for all patients, regardless of geography or resource constraints.
